# The most recent advances in understanding and managing hidradenitis suppurativa

**DOI:** 10.12688/f1000research.26083.1

**Published:** 2020-08-26

**Authors:** Shanthi Narla, Alexis B. Lyons, Iltefat H. Hamzavi

**Affiliations:** 1 1. Multicultural Dermatology Center, Department of Dermatology, Henry Ford Health System, Detroit, MI, USA

**Keywords:** Hidradenitis suppurativa, diet, complementary and alternative medicine, adalimumab, COVID-19, guidelines, international, multidisciplinary, outcome measures, theseus, historic, skin of color, fibroblasts, tunnels, pregnancy, immunomodulatory therapies

## Abstract

Hidradenitis suppurativa (HS) is a chronic, inflammatory, recurrent, and debilitating skin disease of the hair follicle unit that typically develops after puberty. HS has a significant negative impact on both the quality of life (QOL) of patients affected by this disease as well as family members and caregivers. However, the pathogenesis of HS is multifactorial and still remains to be fully elucidated, which makes the development of treatments difficult. The last 10 years have seen a surge in HS research, and many new findings have come to light, yet much more remains to be elucidated. Physicians must employ a multidisciplinary approach to maximally address all facets of HS. Clinical characteristics of the disease that differ between females and males as well as across different races and ethnic groups must be considered. Targeted topical, oral, and injectable therapies continue to be developed for HS as a greater understanding of the pathogenesis is reached. However, randomized controlled trials regarding dietary factors that may contribute to HS are needed to meet our patients’ growing concerns and questions about the role of diet in HS pathogenesis. Finally, improved outcome measures are needed to standardize HS severity and grading between physicians and clinical trials, and a more diverse representation of HS populations is needed in clinical trials.

## Introduction

Hidradenitis suppurativa (HS) is a chronic, inflammatory, recurrent, and debilitating skin disease of the hair follicle unit that typically develops after puberty. The disorder is characterized by comedones, painful inflammatory nodules, abscesses, dermal tunnels, and scarring, with a predilection for intertriginous areas of the body (axillae, inguinal, and anogenital regions) ^1^. HS has a significant negative impact on both the quality of life (QOL) of patients affected by this disease as well as family members and caregivers ^2, 3^. A recent study showed that the negative impact of HS on general health may be higher than that of hypertension, congestive heart failure, type 2 diabetes, myocardial infarction, and depression ^4^. Disease severity (Hurley stage), younger age, diabetes, recent and increasing disease activity, and specific anogenital location have been identified as major aggravating factors of HS ^5^. In addition, chronic pain is experienced in a subset of HS patients that may be a result of recurrent inflammation altering pain perception through central sensitization ^6^. Three key processes have been implicated in the pathogenesis of HS: follicular hyperkeratosis and dilatation, follicular rupture and subsequent inflammatory response, and chronic inflammation characterized by significant tissue architectural changes. Other factors that have been explored as potential causative agents include smoking, mechanical forces, and genetic mutations. Recent investigation has also suggested a potential pathogenic role of microbiome dysfunction ^7^. However, the pathogenesis of HS is multifactorial and still remains to be fully elucidated, which makes the development of treatments difficult.

In this review, we will discuss the most up-to-date emerging concepts in the understanding and management of HS based upon major topics presented at the recent 2019 4 ^th^ annual Symposium on Hidradenitis Suppurativa (SHSA) ^8^. Furthermore, we attempt to predominantly include literature published within the last year (2019–2020) and to also ensure the inclusion and promotion of any novel ideas that may not yet be widely known.

## Epidemiology

### Disease characteristics in female versus male patients

There is a significant variation in the reported prevalence of HS, especially in the difference in prevalence rates between males and females. A recent meta-analysis was performed to determine the pooled overall prevalence of HS, according to geographical region and sex. Subgroup analyses showed that prevalence in males was lower compared to in females in the United States (OR 0.304, 95% CI 0.370–0.439, *P* <0.001). The data demonstrated a lower prevalence in males compared to females in Europe (OR 0.635, 95% CI 0.397–1.1015, *P* = 0.08), but statistical significance was not reached. Finally, there was no difference in prevalence rates in males versus females in the Asia-Pacific region (OR 0.936, 95% CI 0.319–2.751, *P* = 0.78) ^9^.

Jørgensen *et al*. recently conducted a prospective cohort study of 447 consecutive, newly referred patients with HS attending a tertiary dermatological university center in Copenhagen, Denmark. Female patients had a significantly lower age of onset of HS compared to male patients (23.1 [SD 10.3] versus 29.3 [SD 13.6] years, respectively, *P* <0.001). Female patients were also more often obese and had a first-degree relative with HS when compared to male HS patients ^10, 11^. In contrast, females were less likely to smoke and had a lower disease severity measured by Hurley stage, with more females being Hurley stage I (39.4% versus 24.5%) and more males being Hurley stage III (27.0% versus 7.0%, *P* <0.001). Female patients were more likely to have involvement of the groin while males were more likely to have involvement of the gluteal region. Male HS patients had higher inflammatory markers including C-reactive protein (CRP), neutrophil count, and neutrophil/lymphocyte ratio in comparison to female HS patients ^10^.

Apocrine glands were previously thought to be the major targeted skin compartment in HS; however, recent research has focused on the hair follicle as the primary process ^12^. Zaboulis *et al*. recently demonstrated that apocrine glands may indeed be bystanders in HS. Their findings indicated that in contrast to the entire lesional skin in HS patients, an inflammatory signal was not prominent in the apocrine glands. The key dysregulated pathway in female lesional skin was the androgen signaling pathway and in males was the lipid metabolism pathway, reflected in gender-specific transcriptomes ^13^.

### Pregnancy in HS patients

A recent retrospective cohort study revealed that HS patients had a high rate of HS exacerbation during pregnancy and postpartum. Unfortunately, most of these patients did not receive HS-directed medical or procedural treatment during pregnancy and were not seen by dermatologists ^14^. In addition, HS patients have been found to have a higher incidence of anemia compared to controls ^15^, and pregnancy is also associated with anemia. Currently, no standardized guidelines on the management of HS during pregnancy exist. To address this and other knowledge gaps, the National Hidradenitis Suppurativa Pregnancy Registry is being created, which will collect data on HS disease activity and management, including the use of biologic therapies during pregnancy as well as maternal and fetal outcomes ^16^. This registry will supplement the Hidradenitis Suppurativa Prospective Observational Registry and Biospecimen Registry Project (HS PROGRESS) ^17^.

### Pediatric HS

Literature on pediatric HS continues to be scarce. Recently, a cross-sectional, explorative, and descriptive study was conducted based on case note reviews (n = 20), interviews (n = 120), and clinical examination of all pediatric patients (n = 140) undergoing secondary- or tertiary-level care in the Netherlands, Poland, Sweden, Belgium, Spain, France, Italy, Switzerland, Denmark, Slovenia, Qatar, Egypt, Saudi Arabia, Turkey, Tunisia, and South Africa. The cohort of pediatric patients was predominantly female (75.5%) with a median age of 16. A total of 39% reported a first-degree relative with HS. Median BMI was in the 88 ^th^ percentile, and 11% were smokers. Notable comorbidities were acne (32.8%), hirsutism (19.3%), and pilonidal cysts (16.4%). Generally, obesity was prominent in pediatric patients and was related to parent BMI, indicating that there may be benefit in implementing preventative measures in the whole family ^18^. Lastly, the way in which disease was managed in pediatric patients was similar to that in adult patients, but younger patients more commonly exhibited milder disease ^19^.

In comparison, a recent large population-based cohort study showed that both the lightest and the heaviest infants at birth had increased risks of HS. While childhood BMI was also positively associated with a higher risk of HS, returning to normal weight before 13 years of age (around puberty) corresponded to a risk of developing HS similar to that observed in children who were never overweight ^20^.

Finally, Braunberger *et al*. conducted a retrospective chart review at Henry Ford Medical Center demonstrating that boys were more likely to have a prepubescent onset of HS. Girls tended to have postpubescent onset. It was hypothesized that while ovarian hormones may play a large role in adult-onset HS, they may play a less important role in prepubescent disease ^21^.

## Pathophysiology and triggers of HS

### Fibroblasts

Previously, heterogeneous populations of fibroblasts have been identified in other inflammatory disorders such as Crohn’s disease and malignancy and have been shown to contribute to inflammation. Findings in HS are also consistent with these fibroblast subpopulations and their potential contribution to tunnel formation, aggressive squamous cell carcinoma, and the phenotypic presentation of familial HS variants. Potential targets to inhibit the effects of fibroblasts include interleukin (IL)-17, IL-1, Janus kinase–signal transducer and activator of transcription (JAK–STAT), IL-6 receptor, cadherin 11, cyclin-dependent kinases (CDK1, CDK2, CDK4, CDK6), spleen tyrosine kinase (SYK) (reduces IL-6 production via the mitogen-activated protein kinase–protein kinase C [MAPK–PKC] pathway), chemokine ligand 14 (CXCL14), CCN family protein 2 (CCN2), and formyl peptide receptor 2 (FRP2/ALX) as an upstream mediator of STAT1, IL-6, and podoplanin ^22^. A recent case report showed that in two patients with a rare, recalcitrant, ulcerating HS that was not responding to numerous treatments including biologics, prolonged response was seen with a multifactorial regimen including tofacitinib ^23^.

### Diet and weight loss

Recently, a greater number of studies have emerged on the impact of dietary alteration on HS. A cross-sectional survey of 242 patients with HS revealed that 75.8% had eliminated at least one food from their diet. Within this group, 84.6% made changes to multiple food groups. The top five food groups identified were gluten, dairy, refined sugars, tomatoes, and alcohol. A significant portion of the participants (30.9%) reported the dietary change made the HS “much better”. In contrast, some participants did note worsening of their HS when dietary changes were made ^24^.

Furthermore, Marasca *et al*. suggested the inclusion of evaluation of individual nutritional status as an essential part in the management of HS patients ^25^. Barrea *et al*. showed that, compared to a healthy control group, HS patients demonstrated dietary habits tending towards a pro-inflammatory state that was associated with reduced consumption of complex carbohydrates, monosaturated fatty acids, and n-3 saturated fatty acids and a greater intake of saturated fatty acid and n-6 polyunsaturated fatty acids. Moreover, low adherers to the Mediterranean diet had a statistically significantly higher HS Sartorius score ^26^. From our clinical experience, diet is a major concern and focus of HS patients, and large-scale randomized trials are needed to fully elucidate the role of dietary alteration in the management of HS and to better understand its contribution to pathogenesis and physiology. In addition, a symptom and daily food diary may help HS patients identify possible food triggers of disease activity.

Systematic reviews continue to show strong associations between obesity and HS. Bariatric surgery (BS) is a treatment option for morbid obesity and has the potential to ameliorate the effects of chronic inflammatory skin conditions such as HS. However, a retrospective study showed that post-BS, HS patients presented with micro-nutritional deficiencies and insufficient responses to standard first-line antibiotic treatments. Zinc was found to be at significantly lower serum levels in post-BS patients with HS compared to those patients with HS who had not undergone BS ^27^. A recent systematic review also found that a subset of HS patients after BS had a significant increase in panniculus and experienced HS exacerbations in the excess panniculus, requiring excision of loose skin ^28^. These results suggest that post-BS patients may represent a special cohort requiring further medical and surgical intervention to manage HS.

## Treatment options

### Multidisciplinary approach

Garg *et al*. recently conducted a prospective multinational survey of patients between October 2017 and July 2018 to evaluate the unmet needs of HS patients. The results showed that 63.7% of patients visited a physician at least five times before receiving a formal diagnosis of HS. In addition, 54.4% of patients were diagnosed with HS by a dermatologist; however, 20.4%, 10.9%, 4.7%, and 4.2% of patients were diagnosed with HS by primary care doctors, surgeons, obstetrician/gynecologists, and acute care physicians (emergency medicine or hospitalists), respectively. There was also a mean delay in diagnosis of 10.2 ± 8.9 years. Access to dermatology was rated as “difficult” by 37.0%. For symptoms related to HS, 18.3% of participants reported visiting the emergency department more than five times, and 12.5% reported having been hospitalized for HS symptoms more than five times ^29^.

Furthermore, despite a large percentage of HS patients being diagnosed and cared for by healthcare providers other than dermatologists, an EMBASE search of all HS publications over the past 20 years showed that the vast majority of all publications were in dermatology journals (73.3%, 2,106/2,875). This was followed by general medicine (4.9%, 142/2,875) and plastic surgery (3.8%, 108/2,875) journals. Additionally, publications in journals for front-line specialties were sparse (general medicine 4.9%, 142/2,875; obstetrics and gynecology 1.0%, 28/2,875; family medicine 0.4%, 12/2,875; pediatrics 0.2%, 6/2,875; and emergency medicine 0.1%, 4/2,875) ^30^.

Fisher *et al*. analyzed more than 730 posts and 8,500 comments posted on the “Hidradenitis Suppurativa Israel” Facebook group with the aim that, by evaluating the posts’ themes, they would be able to contribute a deeper understanding of HS patient needs and possible ways of engaging those patients. They found that more than 40% of all posts dealt with treatment information requests. There were more than 160 posts, 22.5% of which dealt with emotional aspects, where patients shared their thoughts, hopes, and disappointments, among others. Pessimistic posts, especially those involving the uncertainty in HS, were more frequent (14%) and received over 1,300 comments. They also found that there was a peak of monthly posts in June, July, and August, which may reflect the fact that HS can be exacerbated by the hot weather along with higher humidity ^31^.

**Figure 1.**
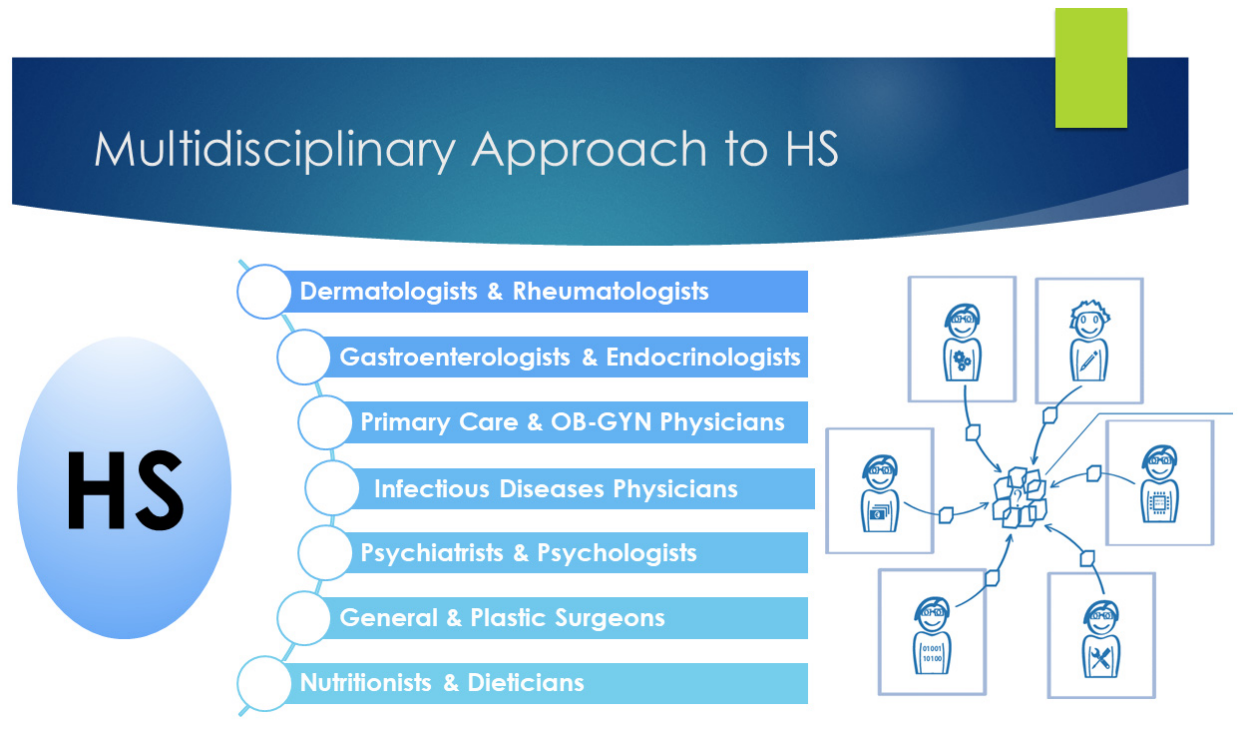
A multidisciplinary approach to hidradenitis suppurativa.

Taking such factors into account, Collier *et al*. emphasized the importance of a comprehensive and multidisciplinary approach for HS patients. Some of their recommendations included the importance of educating front-line providers about HS ^32^. They also emphasized establishing a strong channel of communication between dermatologists and front-line providers, establishing HS specialty clinics, implementing rotations for medical students and residents, creating and moderating virtual or local HS support groups, establishing a multidisciplinary/multispecialty approach to manage the many associated comorbidities ^33^ of HS ( Figure 1), providing education in wound care to both clinic staff and patients, working with infusion clinics to facilitate the administering of intravenous treatment, screening frequently for depression, anxiety, and suicidality, and staying up to date on new therapeutics and ongoing clinical trials for HS ^34^. In addition, cigarette smoking is a known trigger of HS, and thus smoking cessation is recommended as it potentially improves HS as well as other health outcomes ^1^. A recent study was conducted to evaluate the efficacy of a smoking cessation program combining behavioral and pharmacological treatments in HS patients. The results of the study showed that a multidisciplinary intervention including a social service worker, nutritionist, psychologist, and psychiatrist was essential to achieving quitting goals in these patients ^35^.

### Surgery in HS

The primary goals of surgery in HS are removal of the diseased tissue and prevention of recurrence. Recently, Melendez Gonzalez and Sayed further emphasized the importance of surgery in HS. Patients with Hurley stage II and III who have sinus tracts typically require surgery. Both deroofing and local or wide excision procedures have been reported to help significantly. Moreover, medications have a role to play, but as a monotherapy they often have limited ability to achieve optimal outcomes in patients with more advanced disease ^36^. Furthermore, Daveluy highlighted the surgical technique of cryoinsufflation in delineating sinus tracts prior to carbon dioxide laser excision. This involves injecting liquid nitrogen through an ordinary needle directly into HS tracts. Cryoinsufflation could also be used prior to other surgical procedures in HS including deroofing and limited and radical excision. Cryoinsufflation is relatively simple and rapid to perform, and liquid nitrogen is readily available in most dermatology clinics ^37^.

### Adalimumab use during the COVID-19 pandemic

In the setting of the recent COVID-19 pandemic, Blaszczak *et al*. published safety considerations for the use of adalimumab for HS during this time. While there are currently no direct data available for COVID-19 risk in patients with HS, data from the PIONEER I and II phase III clinical trials were extrapolated to highlight the risks of infectious complications in HS patients ^38^. In the PIONEER trials, patients with HS who were taking adalimumab had a modest increased risk of total infections and nasopharyngitis by 2.5% on average, with no difference in the risk of upper respiratory tract infections compared to patients on placebo. However, Blaszczak concluded that it was difficult to extrapolate these data to SARS-CoV-2 infection. Nonetheless, these data may be useful to clinicians for making informed treatment decisions for patients with HS during the ongoing COVID-19 pandemic ^39^. Adalimumab is the only currently FDA-approved systemic medication for the treatment of HS. Other immunomodulatory therapies that have been reported in the treatment of HS are summarized in Table 1 ^40, 41^.

**Table 1.** Summary of immunomodulatory therapies reported in the treatment of hidradenitis suppurativa ^40, 41^.

Monoclonal antibodies	
Mechanism of action	Immunomodulatory therapy
TNF-alpha	• Adalimumab • Etanercept • Infliximab • Golimumab • Certolizumab
IL-1 inhibitors	• Anakinra • Bermekimab • MEDI8968 (NCT 01838499)
IL-17 inhibitors	• Secukinumab • Bimekizumab • Ixekizumab • CJM112 (NCT 02421172)
IL-12/IL-23 inhibitors	• Ustekinumab
IL-23 inhibitor	• Guselkumab
Complement C5a inhibitor	• IFX-1
LFA-1	• Efalizumab
CD20 inhibitors	• Rituximab
**Other systemic immunomodulators**	
Dihydrofolate reductase inhibitor	• Methotrexate
Calcineurin inhibitor	• Cyclosporine
ATRA prodrug: RXR/RAR nuclear transcription factors	• Acitretin • Isotretinoin
Reduction in superoxide production and neutrophil function	• Dapsone
PDE4 inhibitor	• Apremilast
JAK-1 inhibitor	• INCB054707

ATRA, all-trans-retinoic acid; IL, interleukin; JAK, Janus kinase; LFA, lymphocyte function-associated antigen 1; PDE4, phosphodiesterase-4; RAR, retinoic acid receptor; RXR, retinoid-X receptor; TNF, tumor necrosis factor.

### Complementary and alternative medicine use

Complementary and alternative medicine (CAM) modalities are becoming a widely used therapeutic option in patients with HS. A recent study showed that in a cohort of 303 HS patients, 255 patients (84.2%) reported CAM use. The most common reasons for CAM use were reported as “frustration with conventional treatment” and desire to try “new” or “more natural” treatments ^42^. Commonly used CAM products reported by patients included turmeric/curcumin, magnesium sulfate salt bath, and zinc, and patients perceived marijuana, magnesium sulfate bath, and topical cannabidiol oil to be the most helpful. Of the 255 patients who used CAM, 183 said they would recommend CAM use to others, and 166 reported at least mild success with CAM use ^42^. However, despite achieving even mild success with CAM use, it is important to note that only 69.4% reported having informed their HS care provider about CAM use ^42^. Previous studies have shown that patients’ perspectives of how their clinicians would react to their CAM use were generally the most important factor in their openness to discuss their use with physicians ^43^. Clinicians should be encouraged to ask about CAM use in an accepting and non-judgmental way to encourage trust and transparency and to work together with the patient to create the best treatment plan possible.

### Unifying guidelines

Several HS treatment guidelines have been developed since 2015 by various dermatological organizations and expert working groups in North America, South America, and Europe. A review was conducted by Hendricks *et al*. in 2019 to synthesize published guidelines for HS treatment and compare international management recommendations ^44^. The authors found that there was generally agreement on first-line agents, but recommendations for second- and third-line agents varied significantly and were not based on large-scale high-quality trials. The treatments that were strongly and uniformly recommended across guidelines included topical clindamycin, oral tetracyclines, combination clindamycin and rifampin therapy, adalimumab, and wide local excision. However, large-scale randomized controlled trials are lacking for antibiotics and further research is essential to fully elucidate the effects of antibiotics in HS patients. Although targeted biologics were recommended by several guidelines, issues with insurance coverage and a lack of consensus in the use of outcome measurements for HS would make testing and prescribing these treatments difficult. All reviewed guidelines stressed a comprehensive and multidisciplinary approach to the treatment of HS patients ^44^.

Recently, the difficulties in developing unified international guidelines were highlighted by Jemec ^45^. He stressed that although countries working to develop guidelines is commendable, this is happening in parallel, which means that the literature reviewed at the time is similar. With the vast and rapid amount of growing knowledge in HS, it would be more beneficial if countries worked together to create a rotating publication schedule to constantly update guidelines for HS patients and providers based on available evidence ^45^.

## HS unmet needs

### Outcome measures

In a prospective study, 24 patients with HS underwent physical examination by 12 HS experts using nine outcome measure instruments. For all tested outcome measure instruments, the observed intervals for limits of agreements were very wide relative to the ranges of the scales, which implies that substantial changes are needed in clinical practice and research in order to rule out measurement error. Furthermore, the study did not find very good reliability for any included instrument or lesion counts. These results illustrated that even for experienced HS experts, there is difficulty in assessing and grading disease severity of the same lesions in the same patients ^46^.

The Severity and Area Score for Hidradenitis (SASH) was recently developed to assess HS body surface area (BSA) in a clinical trial setting. The inter-rater reliability when using the SASH was 0.60 and improved to 0.75 or 0.76 when the score for BSA for each site was changed from BSA percentage to an ordinal score. In addition, instruments which were dependent on lesion counting, including the modified Sartorius score and inflammatory nodule (AN) count, had lower reliability than SASH. The inter-rater reliability using the Hidradenitis Suppurativa Clinical Response (HiSCR) measure, composed of the AN count, was evaluated in two previous studies and showed an inter-rater reliability of 0.44 (95% CI 0.29–0.63) ^46^ and 0.38–0.67 ^47^. In addition, the International Hidradenitis Suppurativa Severity Score (IHS4) has shown an intraclass correlation coefficient (ICC) of 0.47 (95% CI 0.32–0.65) ^46^. Furthermore, another novel instrument was recently created to measure disease severity called the Hidradenitis Suppurativa Area and Severity Index (HASI), modeled after the Psoriasis Activity and Severity Index. Four classic signs of HS—erythema, thickness, drainage, and tenderness—were selected to be included. The total HASI, HASI without drainage, and HASI without drainage or tenderness all had high inter-rater reliabilities (ICC>0.80) ^48^. These newer scales may significantly improve the ability of clinicians to accurately assess disease status, but further research is needed.

In addition, patient-reported outcome (PRO) measures play an important role in clinical care ^49^. In a recent study, three Patient-reported Outcome Measures Information System (PROMIS) domains (anxiety, pain interference, and depression) were studied in an academic outpatient dermatology department in various patients, including those with HS. In patients with atopic dermatitis (AD) or HS, higher disease severity was associated with high pain interference scores. In HS patients who had documented improvement in treatment between visits, pain interference scores significantly decreased at follow-up visits. These results suggest that utilization of PROMIS domains in routine clinical care in dermatological disorders such as HS may promote further patient-centered care and improve quality of care ^50^.

### Skin of color representation in clinical trials

Using ClinicalTrials.gov and PubMed, Price and colleagues examined the race and ethnicity demographics in phase II and phase III HS treatment trials published from 2000–2019. A total of 15 trials were included in the analysis and among these trials, 669 (68.0%) of the participants were Caucasian and 138 (14.0%) were of African descent. Asians, American Indian or Alaskan Natives, and Native Hawaiian or other Pacific Islanders comprised 29 (2.9%), 3 (0.3%), and 1 (0.1%) participants, respectively. Only 15 participants were reported as Hispanic, as only three trials reported ethnicity data. The remaining 144 (14.6%) participants were recorded as “other/unspecified” (36 self-identified, 108 lacked race reporting). None of the trials included a subanalysis of treatment efficacy based on race or ethnicity ^51^.

Despite adalimumab being the only currently approved systemic medication for the treatment of HS, clinical trials did not sufficiently examine the treatment response in patients of skin of color ^38, 52– 54^. One study was conducted solely in Caucasian and Romany individuals ^52^, while another study consisted of 80–85% Caucasians ^38^. Furthermore, none of the trials reported the percentage of patients who were Hispanic or stratified results by race ^51^. The other systemic biologic agent trials for HS (e.g. etanercept, infliximab, anakinra, and ustekinumab) either did not report race or largely had a Caucasian population ^55– 58^. Moreover, these results suggest that populations represented in clinical trials may not truly reflect the diverse patient populations that are affected by HS and that these medications may work in only certain subsets of HS patients.

## Conclusion

The last 10 years have seen a surge in HS research, and many new findings have come to light, yet much more remains to be elucidated. Physicians must employ a multidisciplinary approach to maximally address all facets of HS. Furthermore, clinical characteristics of the disease that differ between females and males as well as across different races and ethnic groups must be considered. Targeted topical, oral, and injectable therapies continue to be developed for HS as a greater understanding of the pathogenesis is reached. However, randomized controlled trials regarding dietary factors that may contribute to HS are needed to meet our patients’ growing concerns and questions about the role of diet in HS pathogenesis. Finally, improved outcome measures are needed to standardize HS severity and grading between physicians and clinical trials, and a more diverse representation of HS populations is needed in clinical trials.

## Abbreviations

AN, inflammatory nodule; BS, bariatric surgery; CAM, complementary and alternative medicine; HASI, Hidradenitis Suppurativa and Severity Index; HS, hidradenitis suppurativa; ICC, intraclass correlation coefficient; IL, interleukin.
